# The Degree Centrality and Functional Connectivity in Patients With Temporal Lobe Epilepsy Presenting as Ictal Panic: A Resting State fMRI Study

**DOI:** 10.3389/fneur.2022.822253

**Published:** 2022-06-28

**Authors:** Weiwei Chang, Jinping Liu, Liluo Nie, Xiaomin Pang, Zongxia Lv, Jinou Zheng

**Affiliations:** ^1^Department of Neurology, The First Affiliated Hospital of Guangxi Medical University, Nanning, China; ^2^Department of Neurology, The Guilin People's Hospital, Guilin, China

**Keywords:** degree centrality (DC), functional connectivity (FC), TLE, ictal panic, rs-fMRI, middle temporal gyrus (MTG)

## Abstract

**Objectives:**

Ictal panic (IP) can be observed occasionally in patients with temporal lobe epilepsy (TLE). Such descriptions can be found in previous studies, but the mechanism is still not clear and often confused with panic attacks in patients with panic disorder (PD). We try to use imaging methods (resting-state functional magnetic resonance imaging, rs-fMRI) to study the mechanism of this psychiatric comorbidity in patients with TLE.

**Methods:**

Forty right-onset TLE patients were observed, including 28 patients with TLE but without IP and 12 patients with TLEIP along with 30 gender-age matched healthy controls were included. We collected clinical/physiological/neuropsychological and rs-fMRI data. Degree centrality (DC) and functional connectivity (FC) were calculated. For the DC and FC values, analysis of covariance (ANCOVA) was used to find different areas and *t*-tests were used to compare differences between the TLEIP, TLE without IP, and healthy control(HC)groups. The relationship between brain abnormalities and patient characteristics was explored by correlation analyses.

**Results:**

No significant differences in gender and age were found among the three groups, and no significant differences in education level, Montreal Cognitive Assessment (MOCA), Hamilton Depressive Scale (HAMD), Hamilton Anxiety Scale (HAMA), and epilepsy duration (years) between the TLEIP and TLE without IP groups. In addition to fear, other symptoms were observed, including nausea, palpitations, rising epigastric sensation, and dyspnea. There was no correlation between the duration of IP and HAMA. Moreover, all IP durations were <2 min. Compared to the HCs and TLE without IP group, the DC value of the TLEIP group in the left middle temporal gyrus (LMTG) was significantly increased. Compared to the HCs, FC could be found between the LMTG and left inferior temporal gyrus (LITG) in the TLEIP group. In addition, there was FC between the LMTG and cerebellum in the TLEIP group. The difference in the magnitude of FC between the TLEIP vs. HC group was greater than the difference between the TLE vs. HC group.

**Conclusions:**

This study describes brain abnormalities in patients with TLEIP. These results will help to preliminarily understand the mechanism of ictal panic and abnormal functional connection in patients with TLE, and further explore the neuroimaging mechanism of ictal panic in patients with TLE.

## Introduction

Fear is a very distinct and recognized emotion caused by exposure to real or imagined threats (1, 2). The fear response represents a quick repertoire of visceromotor, neuroendocrine, and behavioral mechanisms to the aversive stimuli (3–5). The limbic system is well-recognized as a network of brain structures coordinating such responses (5, 6). The mainstream view is that there are fear circuits in the brain. First, the sensory information for assessing danger is transmitted to the amygdala through the anterior thalamus (7, 8), and when the amygdala perceives the threat, it is immediately activated, triggering a number of different pathways. When the amygdala sends information to the parabrachial nucleus, it can cause shortness of breath (9). If the amygdala sends out information to the lateral hypothalamus, sympathetic nerve activity can be enhanced (10). If the amygdala transmits information to the locus coeruleus, it can cause the secretion and increase of norepinephrine, increase in heart rate and blood pressure, and participate in the fear behavior response (11). If the amygdala transmits information to the paraventricular nucleus axis of the hypothalamus, the secretion of adrenocortical hormone can be increased (12). If the amygdala transmits information to the periaqueductal gray area of the midbrain, it can trigger additional behavioral reactions including defensive behavior and post-escape freezing, which may be related to panic attack symptoms (13–15). But these pathways are not complete. The wider fear network also comprises the prefrontal cortex, anterior cingulate, hippocampus, amygdala, and hypothalamus to learn, store, and evoke fear responses (1). In predisposed individuals exposed to acute or chronic stress, limbic network remodeling may result in the development of psychiatric disorders such as post-traumatic stress disorder, anxiety, and mood disorders (16–18).

Temporal lobe epilepsy (TLE) is a partial form of epilepsy that originates in one or several of the anatomic locations of the temporal lobe, and which can spread through a network of neuronal interconnections to adjacent brain tissue (19). Occasionally, before patients develop secondary generalized tonic-clonic seizure or complex partial seizure, patients with simple partial seizures of temporal lobe origin present with ictal panic which is sometimes treated as panic attacks, a symptom of panic disorder (PD) (20–25). This notion has been supported by some TLE patient studies describing panic disorder during the ictal stage (26–30). Although some studies have focused on the differential diagnosis of TLE with ictal panic and panic attacks, the local brain function mechanisms of TLE with ictal panic are still unclear (31, 32).

Resting-state functional magnetic resonance imaging (rs-fMRI) is now widely used in studies of the human brain. It is an advantageous tool that allows the mapping of regional interactions in the subject's brain when explicit cognitive tasks are not being performed (33, 34). Local dynamics and network functions of the brain can be described by rs-fMRI data, such as degree centrality (DC) and functional connectivity (FC) (35–37).

Degree centrality is proposed to map the degree of functional connectivity inherent in the brain in order to reflect the stability of cortical network structure at the voxel level. FC is the mechanism for the coordination of activity between different neural assemblies in order to achieve a complex cognitive task or perceptual process (38). The two indicators have now been widely used to study the functional modulation of many neuropsychiatric disorders including TLE (39, 40).

Degree centrality describes the importance of individual voxels in the whole brain and can help find areas with abnormal connections with other brain regions. FC can further find the abnormal connection. In this study, we employed an rs-fMRI to explore the brain-functional abnormalities in patients with right-onset TLEIP from different perspectives. Compared to control subjects, we sought to determine whether patients with TLEIP have specific brain-functional abnormalities by using the DC metrics and whether they have abnormal FC. Further, we sought to determine whether these abnormalities were associated with the clinical/physiological/neuropsychological characteristic scores of these patients.

## Materials and Methods

### Participants

All participants were recruited from the epilepsy clinic of the First Affiliated Hospital of Guangxi Medical University. This study was approved by the hospital's Medical Research Ethics Committee. Written informed consent was provided by all participants. Forty patients with TLE were diagnosed by two neuropsychologists according to clinical characteristics, EEGs, and imaging examination. Patients were divided into two groups, TLEIP and TLE without IP groups. In order to reduce the impact on the results, we selected patients with epileptogenic focus on the right as the research subjects.

The inclusion criteria for the TLEIP group involved: (1) Patients with epilepsy who satisfy any two or more of the following conditions: a. The epileptogenic focus was located in the right temporal lobe. b. MRI showed unilateral or bilateral hippocampal atrophy/sclerosis, or other abnormalities in the unilateral or bilateral temporal lobe. c. EEG examination revealed that epileptic discharges originated from the right temporal lobe; (2) Mini-mental state examination (MMSE) scores more than 24, right-handed, 18–50 years, and; (3) TLE history with ictal panic, as a precursor of seizures or as a symptom of seizures.

The following exclusion criteria were used in the TLEIP group: (1) Structural MRI showed other brain structural lesions besides hippocampal atrophy or hippocampal sclerosis; (2) A diagnosis of severe mental or neurological diseases except for ictal panic history; (3) People with alcohol abuse or drug abuse (41), and; (4) Patients who were unable to satisfactorily cooperate and complete all experimental procedures.

The inclusion criteria for the TLE without IP group involved: (1) Patients with epilepsy who satisfy any two or more of the following conditions: a. The epileptogenic focus was located in the right temporal lobe. b. MRI showed unilateral or bilateral hippocampal atrophy/sclerosis, or other abnormalities in the unilateral or bilateral temporal lobe. c. EEG examination revealed that epileptic discharges originated from the right temporal lobe; (2) MMSE scores more than 24, right-handed, 18–50 years, and; (3) TLE history without ictal panic.

The following exclusion criteria were used in the TLE without IP group: (1) Structural MRI showed other brain structural lesions besides hippocampal atrophy or hippocampal sclerosis; (2) A diagnosis of severe mental or neurological diseases; (3) People with alcohol or drug abuse (41), and; (4) Patients who were unable to satisfactorily cooperate and complete all experimental procedures.

Thirty right-handed healthy controls (HCs) without a history of mental or neurological diseases were enlisted from the community. Gender, age, and MMSE scores were matched with those of patients.

### MRI Data Acquisition

MRI data were acquired using an Achieva 3.0-T MRI scanner with a 12-channel head coil (Philips, Amsterdam, The Netherlands). Prior to scanning, each subject was asked to rest for 20 min. During MRI scanning, subjects were instructed to close their eyes, remain conscious, and avoid active thinking. Foam padding was utilized for noise mitigation and to limit head movements. For each subject, resting-state functional imaging was obtained using the echo-planar image (EPI) technique with the following parameters: repetition time (TR) = 2,000 ms, echo time (TE) = 30 ms, 31 slices and 180 volumes, slice thickness = 5 mm, slice gap = 1 mm, voxel size = 3.44 × 3.44 × 6.00 mm, field of view = 220 × 220 mm, flip angle = 90°, scanning time = 360 s.

### Data Preprocessing

Image preprocessing was performed using the Resting-State fMRI Data Analysis Toolkit plus V1.24 (RESTplus V1.24) toolbox (http://restfmri.net/forum/restplus) based on SPM12 (http://www.fil.ion.ucl.ac.uk/spm/software/spm12/), including (1) removing the first 10 time points to make the longitudinal magnetization reach steady state and to let the participant adapt to the scanning environment; (2) slice-timing to correct the differences in image acquisition time between slices; (3) head motion correction; (4) spatial normalization to the Montreal Neurological Institute (MNI) space *via* the deformation fields derived from tissue segmentation of structural images (resampling voxel size = 3 mm × 3 mm × 3 mm); (5) spatial smoothing with an isotropic Gaussian kernel with a full width at half maximum (FWHM) of 6 mm; (6) removing linear trend of the time course; (7) regressing out the head motion effects (using Friston 24 parameter) from the fMRI data, and; (8) band-pass filtering (0.01–0.08 Hz). No participants were excluded from further analysis due to large head motion (more than 3.0 mm of maximal translation in any direction of x, y, or z or 3.0° of maximal rotation throughout the course of scanning)(DC omits step 5).

### DC Calculation

In an undirected graph, degree centrality measures the degree to which one node in the network is associated with all other nodes. For an undirected graph with g nodes, the degree centrality of node i is the total number of direct connections between i and other g-1 nodes, which is represented by a matrix as follows:


$$\begin{array}{l} C_{D}(N_{i})=\sum_{J=1}^{g}x_{ij}(i\neq j) \end{array}$$

Where *C*_*D*_(*N*_*i*_) represents the degree centrality of node i, $\overset{g}{\underset{J=1}{\sum }}x_{ij}$ is used to calculate the number of direct connections between node i and other j(g-1) nodes (i ≠ j, excluding the connection between i and itself; that is, the value of the main diagonal can be ignored). The calculation of *C*_*D*_(*N*_*i*_) is simply to sum the cell values of the corresponding row or column of node i in the network matrix (because undirected relationships form a symmetric data matrix, cells with the same rows and columns have the same values) (42).

### DC and FC Analyses

First, in the DC analysis, the processed data of three groups were analyzed by one-way analysis of covariance (ANCOVA) to find the significant difference regions among groups (GRF correction, one-tailed, voxel level *P* < 0.0005, cluster level *P* < 0.025). Second, (if found) the difference region was made to be a mask and the mask was used to conduct two-sample *t*-tests between each two of the three groups, with the results corrected by GRF (two-tailed, voxel level *P* < 0.001, cluster level *P* < 0.05). Gender, age, and education level were applied as covariates to minimize their potential effects on the analysis.

In the FC analysis, first, through the comparison of DC values, the region with a significant difference between TLEIP and the other two groups is regarded as a region of interest (ROI). In order to explore the difference between TLEIP and TLE without IP, the main parts of the fear circuit: amygdala, hippocampus, parahippocampal gyrus, and thalamus are also taken as ROIs, then voxel-wise FC was performed on the whole brain to calculate the FC between the ROIs and the whole brain. Second, the processed data of the three groups were analyzed by one-way ANCOVA to find the significant difference regions among groups (GRF correction, one-tailed, voxel level *P* < 0.0005, cluster level *P* < 0.025). Third, (if found) the difference region was made to be a mask and the mask was used to conduct two-sample *t*-tests between each two of the three groups, with the results corrected by GRF (two-tailed, voxel level *P* < 0.001, cluster level *P* < 0.05). Gender, age, and education level were applied as covariates to minimize their potential effects on the analysis.

### Statistical Analysis

The clinical/physiological/neuropsychological variables were analyzed using the Statistical Package for the Social Sciences 21.0 (SPSS) (SPSS Inc., Chicago, IL, United States). First, the Shapiro-Wilk test was conducted to determine whether the quantitative data conformed to a normal distribution. Second, if data conformed to a normal distribution, the data of the three groups were statistically analyzed by one-way ANCOVA test, and the data of the two groups were statistically analyzed by an independent *t*-test. For data with a non-normal distribution, the data of the three groups were examined by Kruskal-Wallis nonparametric multiple sample test, and the data of the two groups were examined by the Mann-Whitney U test. Gender differences were tested with the Chi-Square *t*-test.

## Results

### Demographics, Clinical, and Neuropsychological Characteristics

There were no significant differences in gender and age among the TLEIP, TLE without IP, and HC groups. There were no significant differences in education level, MOCA, HAMD, HAMA, and epilepsy duration (years) between the TLEIP and TLE without IP groups (Table 1).

**Table 1:** Comparison of clinical data and neuropsychological scores among the three groups.

	**TLEIP** **(*N* = 12)**	**TLE without IP** **(*N* = 28)**	**HCs** **(*N* = 30)**	**Value**
Gender(M/F)	2/10	5/23	5/25	0.963^a^
Education (years); TLEIP vs. TLE	11.933 ± 2.604	15 (9.75, 16)	17 (16, 17)	H = 24.468 (*P < * 0.001)^b^
				U = 138.000 (*P* > 0.05)^c^
MOCA total score;	27.5 (25.25, 28)	27 (25, 29)	29 (28, 30)	H = 10.619 (*P < * 0.05)^b^
TLEIP vs. TLE				U = 160.000 (*P* > 0.05)^c^
HAMA scores;	5.58 ± 5.035	2.5 (1, 7.7)	0 (0, 2)	H = 18.733 (*P < * 0.001)^b^
TLEIP vs. TLE				U = 140.500 (*P* > 0.05)^c^
HAMD scores;	11.167 ± 7.826	6.607 ± 6.232	1 (0, 3)	H = 27.474 (*P < * 0.001)^b^
TLEIP vs. TLE				t = 1.963 (*P* > 0.05)^d^
Age(years)	29 (25, 30)	30 ± 7.369	25 (23, 30)	H = 2.381 (*P* > 0.05)^b^
Epilepsy duration(years)	14.167 ± 5.638	6 (4, 16)		U = 94.500 (*P* > 0.05)^c^

*Values are mean ± SD*.

*N, number; MOCA, Montreal cognitive assessment; F, female; M, male; HAMD, Hamilton depressive Scale; HAMA, Hamilton anxiety rating scale*.

*^a^Pearson Chi-square tests*.

*^b^Kruskal-Wallis nonparametric multiple sample test*.

*^c^Mann-Whitney U test (two-tailed)*.

*^d^Two-sample t-test*.

**Figure 1:**
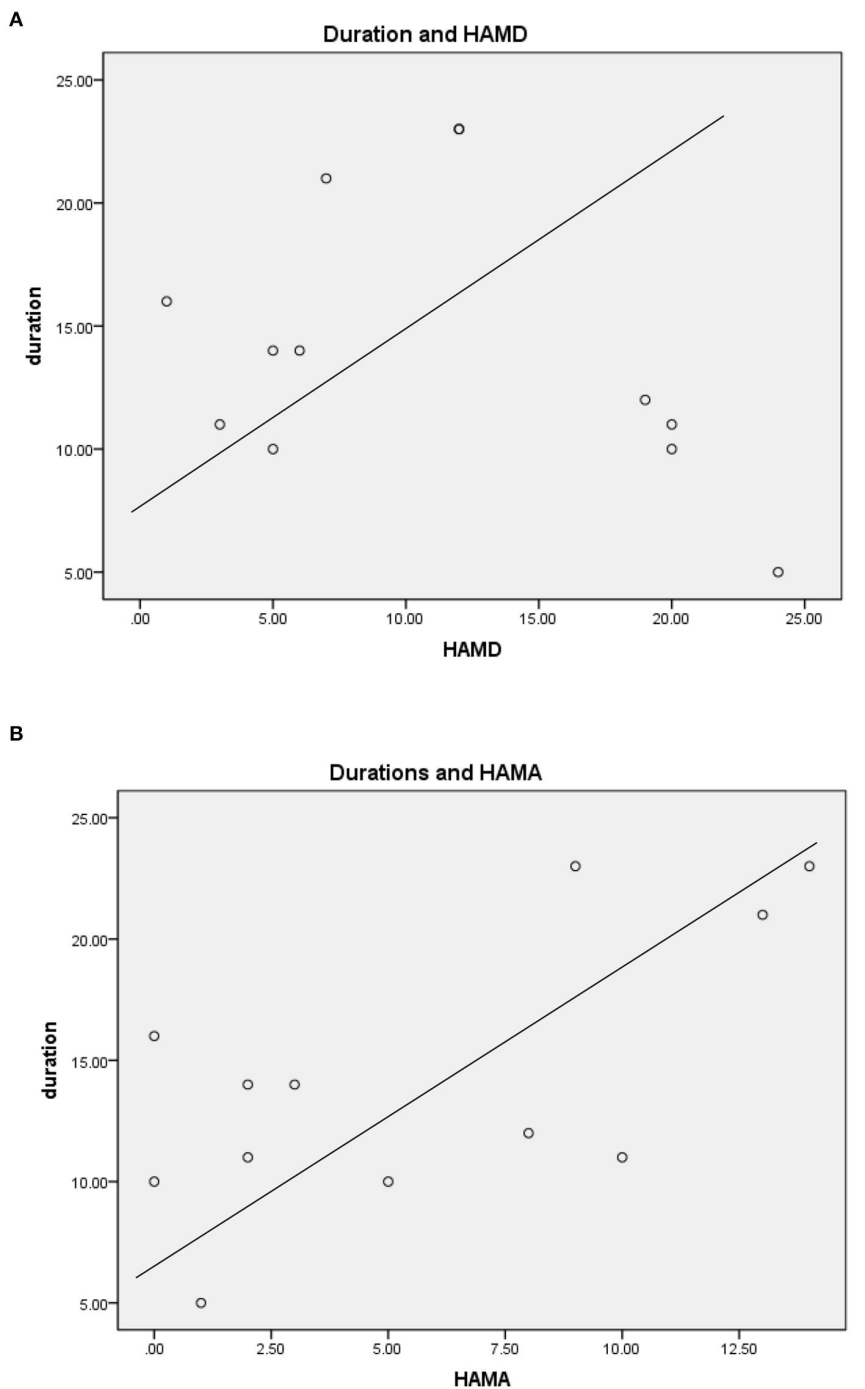
**(A)** Durations of IP symptom and HAMD scores. **(B)** Durations of IP symptom and HAMA scores.

For TLEIP patients, in addition to fear, other symptoms were also observed, including nausea, palpitations, rising epigastric sensation, and dyspnea (43). There were no correlations between duration of IP symptoms and HAMD scores, and there were no correlations between duration of IP symptoms and HAMA scores (Pearson correlation, two-tailed, *p* = 0.659, Figure 1). Moreover, the duration of all IPs were <2 min, and most were <1 min (Table 2).

**Table 2:** Temporal lobe epileptic ictal panic (TLEIP) characteristics.

**Patients**	**Age(years)**	**HAMD**	**Durations of IP symptom (years)**	**HAMA scores**	**Seizures type**	**Symptom**	**Durations of IP (minutes)**
1	46	20	10	0	CP	Fear, nausea	<1
2	45	12	23	9	CP to G	Fear, palpitations, rising epigastric sensation	1–2
3	29	7	21	13	CP to G	Fear	1–2
4	29	1	16	0	SP to G	Fear	<1
5	28	20	11	10	CP	Fear	<1
6	29	5	10	5	SP to G	Fear	<1
7	22	5	14	2	CP to G	Fear	1-2
8	25	24	5	1	CP to G	Fear, palpitations	<1
9	30	3	11	2	SP	Fear	<1
10	23	12	23	14	SP	Fear, dyspnea, palpitations	<1
11	25	19	12	8	CP	Fear	<1
12	30	6	14	3	SP	Fear	<1

*HAMD, Hamilton depressive Scale; CP, complex-partial seizure; SP, simple-partial seizure; G, generalized seizure; HAMA, Hamilton anxiety rating scale*.

### DC and FC Results

**Figure 2:**
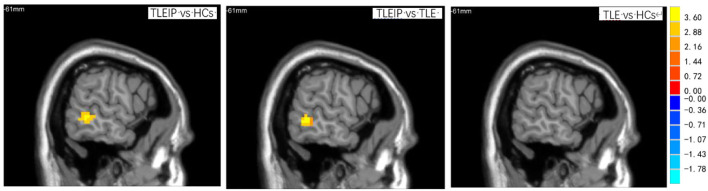
The DC differences among TLEIP, TLE without IP, and HCs. Compared to the HCs and TLE without IP groups, the DC value of the TLEIP group in the left middle temporal gyrus (LMTG) was significantly increased (GRF correction, two-tailed, voxel level *P* < 0.001, cluster level *P* < 0.05).

Compared to the HC and TLE without IP groups, the DC value of the TLEIP group in the left middle temporal gyrus (LMTG) was significantly increased (GRF correction, two-tailed, voxel level *P* < 0.001, cluster level *P* < 0.05) (Table 3 and Figure 2).

**Table 3:** The degree centrality (DC) differences between the TLEIP group, TLE without IP group, and healthy control (HC) group.

**Groups**	**Regions**	**MNI coordinates**	**Cluster voxels**	**T value (peak voxels)**
**DC**				
TLEIP vs. HCs	Middle temporal gyrus_L	(−61, −47,3)	47	4.3149
TLEIP vs. TLE	Middle temporal gyrus_L	(−61, −47,6)	43	4.3360
without IP				
TLE vs. HCs	Negative finding			

**Figure 3:**
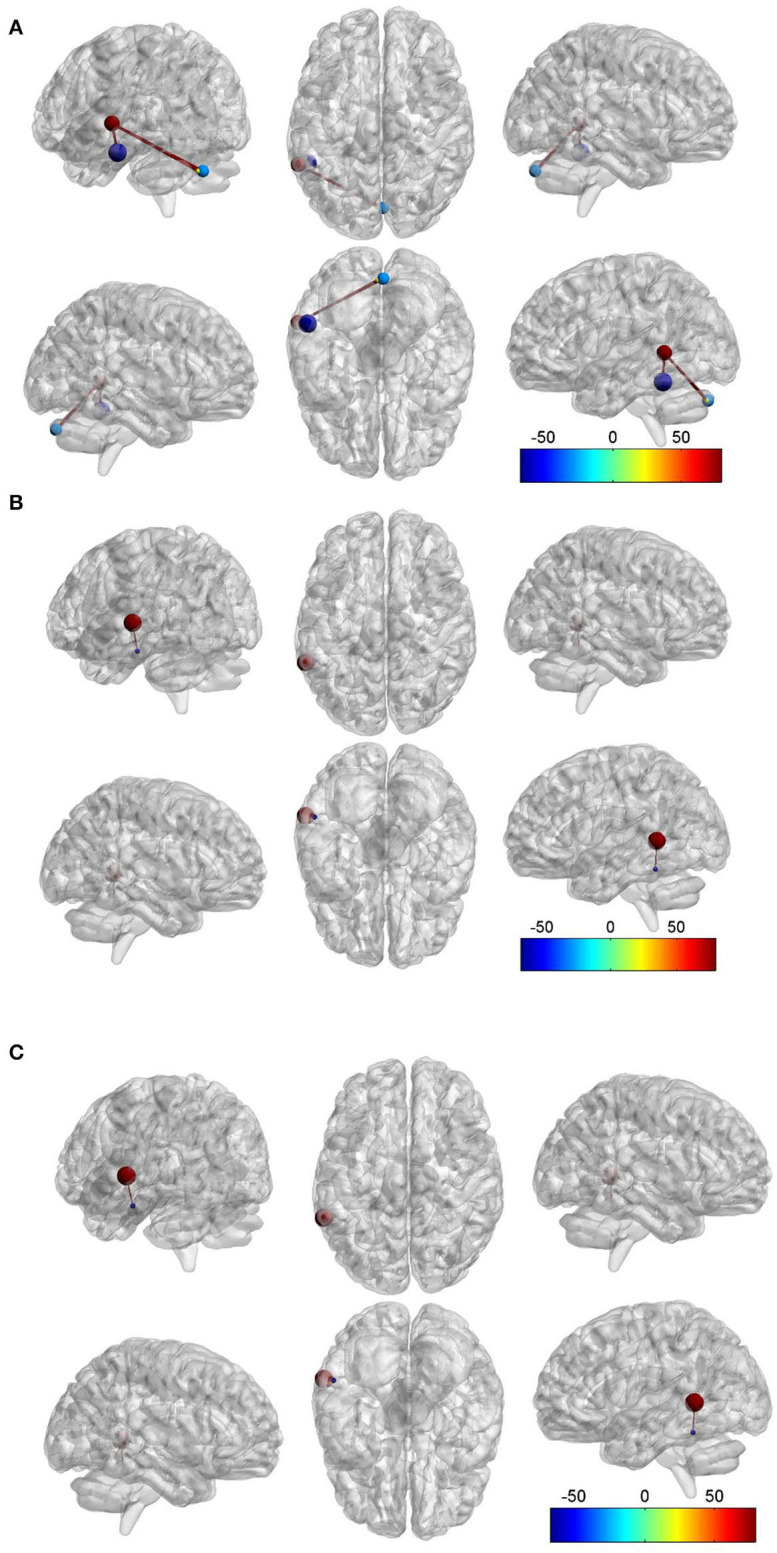
Red ball. the left middle temporal gyrus (LMTG). Purple blue ball, the left inferior temporal gyrus (LITG). Light blue ball, the right cerebellum. Yellow ball, the left cerebellum. Red bar, functional connectivity (FC). **(A)** Compared to the HC group, we found that there was FC between the LMTG and LITG in the TLEIP group, we also found that there were FCs between the LMTG and cerebellum (both left and right) in the TLEIP group. **(B)** Compared to the TLE without IP group, we found that there was FC between the LMTG and LITG in the TLEIP group. **(C)** Compared to the HC group, we found that there was FC between the LMTG and LITG in the TLE without IP group but the area of the LITG in the TLEIP vs. HC group was larger than those in the TLE group vs. HC group. In addition, the difference in the magnitude of FC between the TLEIP vs. HC group was greater than the difference between the TLE without IP vs. HC group. (GRF correction, two-tailed, voxel level *P* < 0.001, cluster level *P* < 0.05).

Compared to the HC group, we found that there was FC between the LMTG and left inferior temporal gyrus (LITG) in the TLEIP group, we also found that there were FCs between the LMTG and cerebellum in the TLEIP group. Although compared to the HC group, we found that there was FC between the LMTG and LITG in the TLE without IP group, the area of LITG in the TLEIP vs. HC group was larger than those in the TLE without IP group vs. HC group. In addition, the difference in the magnitude of FC between the TLEIP vs. HC group was greater than the difference between the TLE without IP vs. HC group. (GRF correction, two-tailed, voxel level *P* < 0.001, cluster level *P* < 0.05) (Figure 3).

## Discussion

In this study, we found that there were no significant differences in HAMA scores between the TLEIP and TLE without IP groups, and there was no significant correlation between the duration of IP symptoms and HAMA scores in the TLEIP group. Therefore, we speculate that there is no correlation between IP and anxiety in patients with TLEIP, which is different from previous studies describing the relationship between anxiety and panic (24, 26, 31, 44). In addition, the duration of IP in patients with TLEIP is different from patients with PD, and the duration of IP in patients with TLEIP is shorter, which is consistent with the observations in a previous study (31). We also found that compared with the HC group, the total MOCA score of the TLE without IP and TLEIP groups was lower, which may be because the education years of the TLE without IP and TLEIP groups were significantly lower than the HCs.

Degree centrality reflects the role and status of voxels in the brain network and represents the most local and directly quantifiable centrality measure. In this study, we found the LMTG exhibited increased DC, which indicated increased importance of this region in the brain of patients with TLEIP. The LMTG was involved in several functions, including semantic processing, sentence understanding, word generation, action observation, complex sound processing, logical reasoning, and dynamic facial expression recognition (45–52). Generalized social anxiety disorder (GSAD) is one of the most common anxiety disorders and mainly involves a notable fear and avoidance of most social or performance situations. Yuan et al. found that the DC value of the LMTG in patients with GSAD before group cognitive behavior therapy (GCBT) is increased than the DC value of the LMTG in patients with GSAD after GCBT. This may suggest the role of LMTG in the disease with fear comorbidity (53). Zhao and colleagues found that fearful faces evoked greater activity in the LMTG (54). Moreover, by using functional MRI, Takano et al. (55) investigated common and distinct neural responses to experiences of positive- and threat-awe, elicited by watching awe-inspiring videos, and found that both awe experiences deactivated the LMTG in contrast to the control conditions (positive-awe vs. amusement; threat-awe vs. fear), which meant the fear experience activated the LMTG. Geng et al. (56) found that high trait anxious individuals showed significantly increased activation in the middle temporal gyrus (MTG) during anticipation of an uncertain threat compared to the certain condition. Additionally, a recent meta-analytic work (57) found that the LMTG was activated when fear stimulation was given to adults with childhood trauma. Thus, we speculate that the increased DC in the LMTG may indicate increased FC with the fear circuit, and may explain the panic attack of some patients with TLEIP when stimulated by the external environment, such as harsh sounds and scary pictures.

Harnett et al. (58) used a temporal Pavlovian conditioning procedure to investigate brain activity that mediates the formation of temporal associations. During fixed interval trials, greater conditioned fMRI signal responses were observed within the dorsolateral prefrontal cortex, inferior parietal lobule, inferior and middle temporal cortex, hippocampus, and amygdala. They thought these brain regions constitute a neural circuit that encodes the temporal information necessary for Pavlovian fear conditioning. The result is consistent with the enhanced connection between MTG and ITG found in our study. Eser et al. (59) studied the functional correlates of cholecystokinin tetrapeptide (CCK-4)-induced experimental panic in healthy volunteers by means of fMRI and ROI analysis of the amygdala. They found CCK-4-induced experimental panic was accompanied by a robust activation (random-effects analysis, *P* < 0.00001, uncorrected for multiple testing) in the LMTG and cerebellum. In contrast, random-effects group analysis for placebo and anticipatory anxiety (AA) using the same level of significance generated no significant results. In this study, we found that the FC between the LMTG and the cerebellum was strengthened in patients with TLEIP, which also verified this result. Although no local brain and FC abnormalities were found in the amygdala, hippocampus, parahippocampal gyrus, and thalamus, we speculate that the mechanism of TLEIP may not be completely the same as the mechanism of PD. This may mainly be due to the transient and recoverable incomplete activation of the fear circuits caused by the epileptiform discharge of the local epileptogenic focus in the temporal lobe (60–62), and it may be related to some non-classical fear circuit brain regions, such as the increased connection between the LMTG and the fear circuit.

This study has some limitations that need to be recognized:

- due to the requirements of clinical ethics, all patients had been treated with anti-epilepsy drugs (AEDs)
- it was difficult to collect patients with TLEIP, a large number of samples were not included in this study
- lack of horizontal comparison with patients with PD
- participants are patients with right-onset TLE, more patients with left-onset TLE need to be collected to have a further study.

In the future, we will collect more patients with left-onset TLE and patients with PD for study to further explore the mechanism of fear in the functional brain network.

## Data Availability Statement

The original contributions presented in the study are included in the article/supplementary material, further inquiries can be directed to the corresponding author.

## Ethics Statement

The studies involving human participants were reviewed and approved by the First Affiliated Hospital's Medical Research Ethics Committee of Guangxi Medical University. The patients/participants provided their written informed consent to participate in this study. Written informed consent was obtained from the individual(s) for the publication of any potentially identifiable images or data included in this article.

## Author Contributions

WC is responsible for experimental design. JZ is responsible for providing overall ideas. ZL is responsible for instrument operation. XP is responsible for data analysis. JL and LN is responsible for data collection. All authors contributed to the article and approved the submitted version.

## Funding

This research is supported by the National Natural Science Foundation of China (contract authorization number: 81560223) and Guangxi Postgraduate Education Innovation Plan (authorization number: YCBZ2019042).

## Conflict of Interest

The authors declare that the research was conducted in the absence of any commercial or financial relationships that could be construed as a potential conflict of interest.

## Publisher's Note

All claims expressed in this article are solely those of the authors and do not necessarily represent those of their affiliated organizations, or those of the publisher, the editors and the reviewers. Any product that may be evaluated in this article, or claim that may be made by its manufacturer, is not guaranteed or endorsed by the publisher.
